# Direct involvement of *ombB*, *omaB*, and *omcB* genes in extracellular reduction of Fe(III) by *Geobacter sulfurreducens* PCA

**DOI:** 10.3389/fmicb.2015.01075

**Published:** 2015-10-01

**Authors:** Yimo Liu, James K. Fredrickson, John M. Zachara, Liang Shi

**Affiliations:** Pacific Northwest National Laboratory, Richland, WAUSA

**Keywords:** Fe(III) reduction, *Geobacter*, porin-cytochrome, trans-outer membrane protein complex, extracellular electron transfer

## Abstract

The tandem gene clusters *orfR*-*ombB-omaB-omcB* and *orfS*-*ombC-omaC-omcC* of the metal-reducing bacterium *Geobacter sulfurreducens* PCA are responsible for trans-outer membrane electron transfer during extracellular reduction of Fe(III)-citrate and ferrihydrite [a poorly crystalline Fe(III) oxide]. Each gene cluster encodes a putative transcriptional factor (OrfR/OrfS), a porin-like outer-membrane protein (OmbB/OmbC), a periplasmic *c*-type cytochrome (*c*-Cyt, OmaB/OmaC) and an outer-membrane *c*-Cyt (OmcB/OmcC). The individual roles of OmbB, OmaB and OmcB in extracellular reduction of Fe(III), however, have remained either uninvestigated or controversial. Here, we showed that replacements of *ombB*, *omaB*, *omcB*, and *ombB-omaB* with an antibiotic gene in the presence of *ombC-omaC-omcC* had no impact on reduction of Fe(III)-citrate by *G. sulfurreducens* PCA. Disruption of *ombB*, *omaB*, *omcB*, and *ombB-omaB* in the absence of *ombC-omaC-omcC*, however, severely impaired the bacterial ability to reduce Fe(III)-citrate as well as ferrihydrite. These results unequivocally demonstrate an overlapping role of *ombB-omaB-omcB* and *ombC-omaC-omcC* in extracellular Fe(III) reduction by *G. sulfurreducens* PCA. Involvement of both *ombB-omaB-omcB* and *ombC-omaC-omcC* in extracellular Fe(III) reduction reflects the importance of these trans-outer membrane protein complexes in the physiology of this bacterium. Moreover, the kinetics of Fe(III)-citrate and ferrihydrite reduction by these mutants in the absence of *ombC-omaC-omcC* were nearly identical, which suggests that absence of any protein subunit eliminates function of OmaB/OmbB/OmcB protein complex. Finally, *orfS* was found to have a negative impact on the extracellular reduction of Fe(III)-citrate and ferrihydrite in *G. sulfurreducens* PCA probably by serving as a transcriptional repressor.

## Introduction

*Geobacter* sp. play critical roles in global cycling of carbon, iron (Fe), manganese (Mn), and other elements. They can oxidize organic matter intracellularly and then transfer the released electrons to the terminal electron acceptors, such as Fe(III) and Mn(IV) oxides that are external to the bacterial cells. Because of their extracellular electron transfer capability, *Geobacter* sp. have been harnessed for the bioremediation of metal and organic contaminants in the subsurface sediments and for bioenergy production (Lovley et al., 2004, 2011).

To reduce extracellular Fe(III) and Mn(IV) oxides, *Geobacter* sp. transfer electrons from the quinone/quinol pool in the cytoplasmic or inner membrane, through the periplasm and across the outer membrane to the oxide surfaces directly via *Geobacter* nanowires and/or redox proteins, such as *c*-type cytochromes (*c*-Cyt; Weber et al., 2006; Shi et al., 2007, 2009; Bird et al., 2011; Strycharz-Glaven et al., 2011; Bond et al., 2012; Snider et al., 2012). The outer membrane of Gram-negative bacteria, however, is a physical barrier for electron conductance (Shi et al., 2012a). To overcome this barrier, *Geobacter sulfurreducens* PCA employ the porin-cytochrome (Pcc) trans-outer membrane protein complexes for electron conductance across the outer membrane (Liu et al., 2014; Shi et al., 2014). The characterized Pcc protein complexes consist of a porin-like outer-membrane protein (OmbB or OmbC), a periplasmic 8-heme *c*-Cyt (OmaB or OmaC) and an outer-membrane 12-heme *c*-Cyt (OmcB or OmcC). The Pcc protein complexes have been isolated from the membrane fraction of *G. sulfurreducens* PCA cultured with Fe(III)-citrate as the terminal electron acceptor. After they were reconstituted in proteoliposomes, the isolated Pcc protein complexes transferred electrons from methyl viologen inside the proteoliposomes, across the lipid-bilayer to the external Fe(III)-citrate and ferrihydrite [a poorly crystalline Fe(III) oxide] (Liu et al., 2014). It is proposed that OmbB or OmbC serves as a scaffold through which OmaB or OmaC and OmcB or OmcC are inserted to form a heme-based electron conduit of sufficient length to span the entire width of outer membrane. This model is similar to that proposed for MtrABC trans-outer membrane protein complex of the metal-reducing bacterium *Shewanella oneidensis* MR-1(Hartshorne et al., 2009; Richardson et al., 2012; White et al., 2013; Liu et al., 2014). In *S. oneidensis* MR-1, MtrABC complex is responsible for trans-outer membrane electron transfer during extracellular reduction of Fe(III) oxides and can transfer electrons directly to Fe(III) oxides at rates sufficient to support *in vivo* anaerobic respiration of *S. oneidensis* MR-1 (Hartshorne et al., 2009; White et al., 2013). It should be pointed out that the Pcc proteins and Mtr proteins are phylogenetically unrelated and they appear to have evolved independently to the similar functions (Liu et al., 2014; Shi et al., 2014).

**FIGURE 1:**
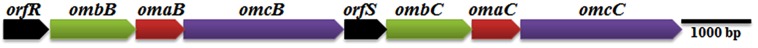
**The *orfR* gene clusters of *Geobacter sulfurreducens* PCA.** The genes encoding transcriptional factors, porin-like outer-membrane proteins, the periplasmic *c*-type cytochromes and the outer-membrane *c*-type cytochromes are labeled in black, green, red, and purple, respectively.

The genes encoding Pcc proteins are clustered in the same region (i.e., pcc gene cluster) of bacterial genomes. The pcc gene clusters are found in the genomes of all sequenced *Geobacter* sp. and 11 other bacteria from six different phyla, which reflect the importance of Pcc protein complexes in trans-outer membrane electrons transfer by the Gram-negative bacteria (Shi et al., 2014). *G. sulfurreducens* PCA possesses four pcc gene clusters and two of which, *ombB-omaB-omcB* and *ombC-omaC-omcC*, are directly involved in extracellular reduction of Fe(III) (Liu et al., 2014; Shi et al., 2014). The *ombB-omaB-omcB* and *ombC-omaC-omcC* are part of tandem gene clusters, which also include *orfR* and *orfS* that encode putative transcriptional factors (**Figure 1**; Leang et al., 2003; Leang and Lovley, 2005; Liu et al., 2014; Shi et al., 2014). At the amino acid sequence level, OmbB/OmbC and OmaB/OmaC are 100% identical, respectively; while OrfR/OrfS and OmcB/OmcC are 99 and 71% identical, respectively. Thus, *orfR*-*ombB-omaB-omcB* and *orfS*-*ombC-omaC-omcC* are a result of gene duplication (Leang et al., 2003; Liu et al., 2014; Shi et al., 2014).

Previously, we found that replacement of *ombB-omaB-omcB* or *ombC-omaC-omcC* with an antibiotic resistant gene had little or no impact on Fe(III)-citrate reduction and limited impacts on ferrihydrite reduction by *G. sulfurreducens* PCA, while disruption of *ombB-omaB-omcB*-*orfS*-*ombC-omaC-omcC* significantly impaired the ability of *G. sulfurreducens* PCA to reduce Fe(III)-citrate and ferrihydrite. All these results demonstrate the direct involvement of both *ombB-omaB-omcB* and *ombC-omaC-omcC* in extracellular reduction of Fe(III)-citrate and ferrihydrite (Liu et al., 2014). However, the roles of individual genes of these clusters, such as *ombB* and *omaB*, in extracellular reduction of Fe(III) had remained largely uninvestigated. Moreover, the role of *omcB* in Fe(III) reduction had remained controversial (Leang et al., 2003; Liu et al., 2014). In contrast to our previous results in *G. sulfurreducens* PCA (Liu et al., 2014), disruption of *omcB* in the presence of *omcC* greatly impaired the bacterial ability to reduce Fe(III)-citrate by *G. sulfurreducens* DL-1, which suggests that OmcC is not involved in Fe(III)-citrate reduction (Leang et al., 2003). Moreover, OmcB was once believed to mediate electron conductance across the outer membrane by itself because it was deeply embedded in the outer membrane (Qian et al., 2007; Butler et al., 2009). To further clarify their roles, we conducted detailed characterizations of *ombB*, *omaB*, and *omcB* with regards to extracellular reduction of Fe(III)-citrate and ferrihydrite by *G. sulfurreducens* PCA.

## Materials and Methods

### Bacterial Growth, Mutant Construction, and Gene Cloning

*Geobacter sulfurreducens* PCA (ATCC^®^ 51573^TM^) was routinely cultured in the medium with 10 mM acetate as an electron donor and 40 mM fumarate as an electron acceptor prior to construction of gene replacement mutants. The gene replacement mutants and related complement strains were constructed by using established protocols (Coppi et al., 2001; Leang et al., 2003; Lloyd et al., 2003; Rollefson et al., 2009; Liu et al., 2014). Briefly, the genomic DNA of *G. sulfurreducens* PCA was purchased from ATCC (Manassas, VA, USA), which served as a template for PCR amplification of respective PCR fragments that flanked the target genes. The kanamycin and chloramphenicol resistance genes were amplified with pBBR1-MCS2 and pACYC184 as templates, respectively (Chang and Cohen, 1978; Kovach et al., 1995). The PCR fragments that flanked the targeted genes and PCR-amplified kanamycin or chloramphenicol resistance gene were mixed and then served as the templates for the second round PCR amplification of the fragments that contained respective gene replacement mutants. These fragments from the second round PCR were electroporated into the target cells separately to make individual gene replacement mutant via double-homologous recombination between the flanking regions of the PCR fragments and the flanking regions of the target genes on the chromosome (Coppi et al., 2001; Lloyd et al., 2003).

For cloning, the target genes were separately amplified by PCR with their respective primers. After treatment with restriction enzymes, such as BamHI, EcoRI, HindIII, SpeI, and XhoI (New England Biolabs, Ipswich, MA, USA), the PCR fragments were cloned into pBBR1-MCS5 by using the Fast-link DNA ligation kit (Epicenter, Madison, WI, USA; Kovach et al., 1995). After verification by sequencing, the cloned genes were introduced to their respective mutants by conjugation via *Escherichia coli* strain WM3064 (Coppi et al., 2001). All gene replacement mutants and complement strains were confirmed by PCR amplifications of the disrupted regions of bacterial genome and the cloned genes in a plasmid, respectively. Bacterial strains, plasmids, and oligonucleotide primers used in this study are listed in Supplementary Table S1. The procedures for SDS-PAGE and heme staining were described previously (Thomas et al., 1976; Shi et al., 2006).

### Fe(III) Reduction

Amorphous 2-line ferrihydrite was synthesized by hydrolysis of Fe(NO_3_)_3_ solution at pH 7 at room temperature. The synthesis was conducted inside an anaerobic chamber to prevent any CO_2_ contamination (100% N_2_, Innovative Technology, Inc., Amesbury, MA, USA). Briefly, 140 g of Fe(NO_3_)_3_⋅9H_2_O was dissolved in 700 mL of ddH_2_O in a 1 L Teflon bottle. The pH of the Fe(NO_3_)_3_ solution was slowly adjusted with 2 M NaOH solution until pH 7, which yielded a dark brown suspension. After overnight equilibration under a N_2_ atmosphere, the suspension pH was still 7. The suspension was centrifuged and resuspended in ddH_2_O, and this washing procedure was repeated for eight times (Schwertman and Cornell, 2000; Shi et al., 2012b). The synthesized two-line ferrihydrite was characterized using transmission electron microscopy (TEM, Jeol JEM 2010 high-resolution TEM, Peabody, MA, USA) and powder X-ray diffraction (XRD, Philips PW 3040/00 X’pert MPD system, Westborough, MA, USA). Fe(III)-citrate was prepared as described previously (Wang et al., 2008). For Fe(III)-reduction assays, all *Geobacter* strains were pre-cultured in the medium with fumarate as an electrons acceptor. Antibiotics were used at 200 μg/ml for kanamycin and 10 μg/ml for chloramphenicol and gentamicin. Reduction of 50 mM of Fe(III)-citrate or two-line ferrihydrite was carried out at 30°C with *Geobacter* cells at starting OD_600_ of 0.05 in the absence of antibiotic (Leang et al., 2003; Rollefson et al., 2009; Liu et al., 2014). All procedures were performed in an anaerobic chamber (Coy Laboratory Products Inc., Grass Lake, MI, USA) that was filled with 5% H_2_, 20% CO_2_, and 75% N_2_. The reduced Fe(II) was measured with a ferrozine assay (Stookey, 1970), and total Fe was determined with inductively coupled plasma emission spectroscopy (Perkin-Elmer, Waltham, MA, USA).

## Results

### Characterization of *ombB*, *omaB*, and *omcB* in the Presence of *ombC-omaC-omcC*

**FIGURE 2:**
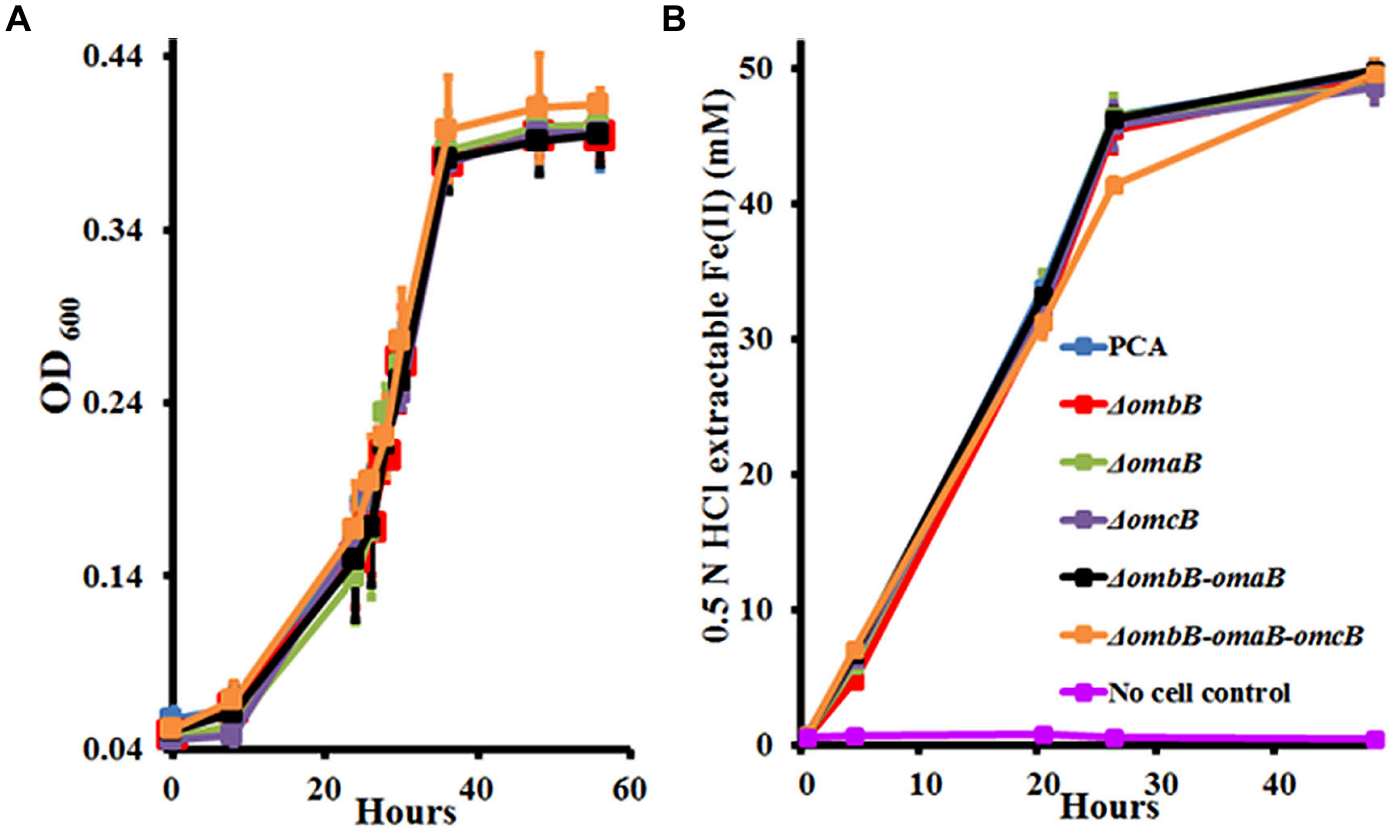
**Characterization of *ombB*, *omaB*, and *omcB* in the presence of *ombC-omaC-omcC*. (A)** Growth on fumarate. **(B)** Fe(III)-citrate reduction. The curves are labeled in the same way in **(A,B)**, except that no cell control is omitted in **(A)**. The values plotted at each time point are the average OD_600_ **(A)** and 0.5 N HCl extractable Fe(II) **(B)** measured for each strain from triplicate assays, respectively, and error bars are standard deviations. For points without error bar, the error was smaller than the symbol.

To characterize their roles, we first replaced *ombB, omaB*, *omcB*, and *ombB-omaB* with the kanamycin or chloramphenicol resistance gene. Following verification (Supplementary Figure S1A), the resulting mutants were tested for their growth with fumarate or Fe(III)-citrate as the terminal electron acceptor. For comparison, a previously made Δ*ombB-omaB-omcB* was included. As shown in **Figure 2**, replacements of *ombB, omaB*, *omcB*, and *ombB-omaB* had no impact on bacterial growth with fumarate or reduction of Fe(III)-citrate. Consistent with our previous results, even replacement of the entire *ombB-omaB-omcB* gene cluster only slightly decreased the ability of *G. sulfurreducens* PCA to reduce Fe(III)-citrate at 24 h. Given that both *ombB-omaB-omcB* and *ombC-omaC-omcC* are involved in Fe(III)-citrate reduction (Liu et al., 2014), the lack of apparent phenotype of these mutants in reducing Fe(III)-citrate suggests an overlap function between *ombB-omaB-omcB* and *ombC-omaC-omcC* in Fe(III) reduction, which makes it difficult to accurately evaluate the functions of *ombB*, *omaB*, and *omcB* in the presence of *ombC-omaC-omcC*.

### Characterization of *ombB*, *omaB*, and *omcB* in the Absence of *ombC-omaC-omcC*

**FIGURE 3:**
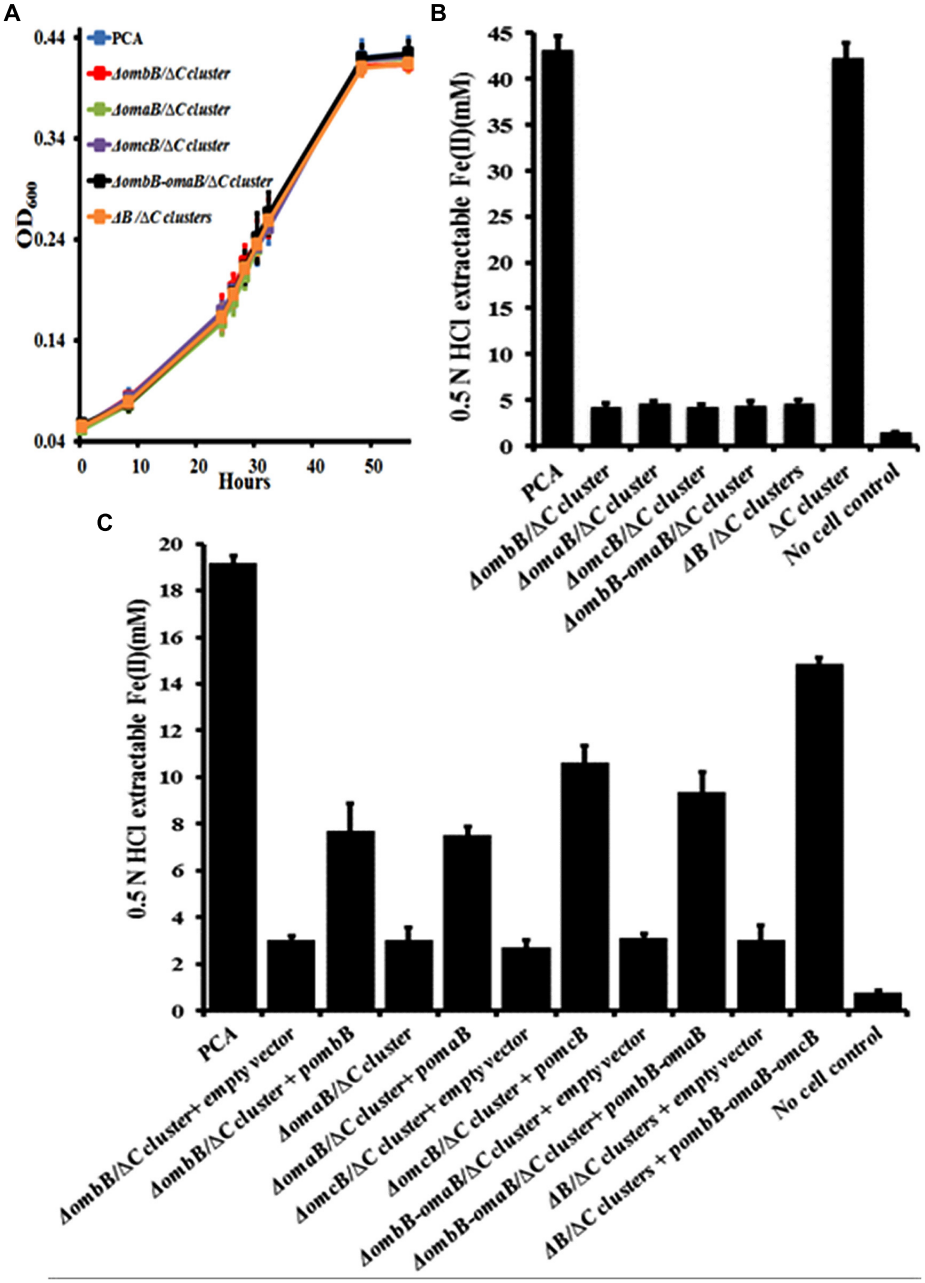
**Characterization of *ombB*, *omaB*, and *omcB* in the absence of *ombC-omaC-omcC*. (A)** Growth on fumarate. **(B)** Fe(III)-citrate reduction at 26 h. **(C)** Ferrihydrite reduction at 360 h. The values plotted at each time point are the average OD_600_ **(A)** and 0.5 N HCl extractable Fe(II) **(B,C)** measured for each strain from triplicate assays, respectively, and error bars are standard deviations. For points without error bar, the error was smaller than the symbol **(A)**.

To avoid any interference of *ombC-omaC-omcC*, we replaced *ombB, omaB*, *omcB*, *ombB-omaB*, and *ombB-omaB-omcB* with a chloramphenicol resistance gene in a previously constructed Δ*ombC-omaC-omcC* (Supplementary Figure S1B) (Liu et al., 2014). Replacements of these genes had no impact on bacterial growth with fumarate as the terminal electron acceptor (**Figure 3A**), but significantly lowered the bacterial reduction of Fe(III)-citrate (**Figure 3B**). At 26 h, *G. sulfurreducens* PCA and Δ*ombC-omaC-omcC* reduced 43.3 ± 1.4 and 42.4 ± 1.5 mM Fe(III)-citrate (*n* = 3), respectively, while Δ*ombB*/Δ*ombC-omaC-omcC* (or Δ*C* cluster), Δ*omaB*/Δ*C* cluster, Δ*omcB*/Δ*C* cluster, Δ*ombB-omaB*/Δ*C* cluster, and Δ*B*/Δ*C* clusters had a decrease of at least 89% of the wild type’s ability to use Fe(III)-citrate as the terminal electron acceptor (**Figure 3B**). Moreover, the kinetics of Fe(III)-citrate reduction by these newly constructed mutants were nearly identical.

Apparently varied expression levels of heme-containing of OmcB or OmaB were observed in different gene disruption mutants (Supplementary Figures S1 and S2). To test any polar effect of gene-replacement, we cloned *ombB, omaB*, *omcB*, *ombB-omaB*, and *ombB-omaB-omcB* and introduced the cloned genes into their respective mutants. Empty vector was also introduced into the mutants and resulting strains served as controls. Addition of empty vector had little impact on Fe(III) reduction as the rates for reducing Fe(III)-citrate were nearly identical for those with or without empty vector (**Figure 3B**; Supplementary Figure S2). Compared to the controls that were introduced with the empty vector, complemented strains exhibited 2- to 5-fold increase in reducing Fe(III)-citrate at 48 h (Supplementary Figure S2). Because complement *omaB* and *omcB* displayed varied levels of expressed proteins (Supplementary Figure S3), the observed difference in Fe(III)-citrate reduction by the complement strains is most likely due to different expression levels of complement genes. All these results consistently show that the phenotypes of Fe(III)-citrate reduction exhibited by these mutants are unlikely attributable to any secondary effect of disrupting these genes. We then tested the impacts of disrupting *ombB, omaB*, *omcB*, *ombB-omaB*, and *ombB-omaB-omcB* on the ability of *G. sulfurreducens* PCA to reduce ferrihydrite with these strains.

As shown in **Figure 3C**, at 360 h, *G. sulfurreducens* PCA reduced 19.2 ± 1.2 mM ferrihydrite (*n* = 3), however, Δ*ombB*/Δ*C* cluster, Δ*omaB*/Δ*C* cluster, Δ*omcB*/Δ*C* cluster, Δ*ombB-omaB*/Δ*C* cluster, and Δ*B*/Δ*C* cluster, which all contained the empty vector, only reduced 2.7 ± 0.3 to 3.1 ± 0.2 mM ferrihydrite (*n* = 3). Similar to the results of Fe(III)-citrate reduction, the kinetics of ferrihydrite reduction by these mutants were nearly identical and complements with their respective genes increased the extent of ferrihydrite reduction from 2.5- to 4.9-fold.

### Negative Role of *orfS* in Fe(III) Reduction

**FIGURE 4:**
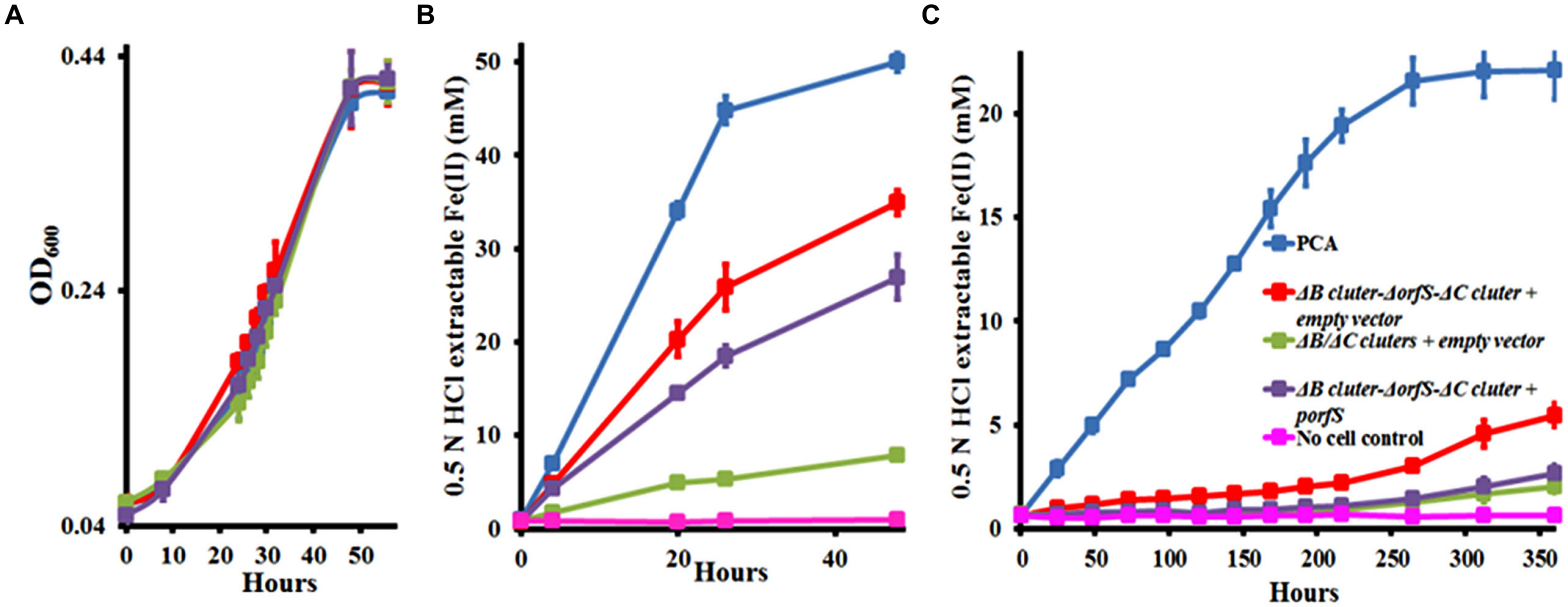
**Characterization of *orfS*. (A)** Growth on fumarate. **(B)** Fe(III)-citrate reduction. **(C)** Ferrihydrite reduction. The curves are labeled in the same way in **(A–C)**, except that no cell control is omitted in **(A)**. The values plotted at each time point are the average OD_600_ **(A)** and 0.5 N HCl extractable Fe(II) **(B,C)** measured for each strain from triplicate assays, respectively, and error bars are standard deviations. For points without error bar, the error was smaller than the symbol.

During the measurements, we noticed that the Δ*B*/Δ*C* clusters constructed in this study reduced much less Fe(III)-citrate than the Δ*ombB-omaB-omcB*-*orfS*-*ombC-omaC-omcC* prepared previously (**Figure 4B**). The only difference between these two mutants is the presence of *orfS* in the Δ*B*/Δ*C* clusters, suggesting that the difference is related to the function of *orfS*.

To further explore the *orfS* function, we complemented Δ*ombB-omaB-omcB*-*orfS*-*ombC-omaC-omcC* with the cloned *orfS*. An empty vector was also introduced into Δ*B*/Δ*C* clusters and Δ*ombB-omaB-omcB*-*orfS*-*ombC-omaC-omcC* and resulting strains served as controls. All these strains and *G. sulfurreducens* PCA showed the nearly identical growth pattern with fumarate as the terminal electron acceptor (**Figure 4A**). However, they reduced Fe(III)-citrate differently. At 48 h, *G. sulfurreducens* PCA and Δ*B*/Δ*C* clusters with the empty vector reduced 50.0 ± 1.0 and 7.8 ± 0.4 mM Fe(III)-citrate (*n* = 3), respectively, while Δ*ombB-omaB-omcB*-*orfS*-*ombC-omaC-omcC* with the empty vector and Δ*ombB-omaB-omcB*-*orfS*-*ombC-omaC-omcC* with *orfS in trans* reduced 34.0 ± 1.3 and 26.9 ± 2.4 mM Fe(III)-citrate (*n* = 3), respectively. Thus, complement of Δ*ombB-omaB-omcB*-*orfS*-*ombC-omaC-omcC* with *orfS* further decreased Fe(III)-citrate reduction by 23% (**Figure 4B**). Similarly, complement of Δ*ombB-omaB-omcB*-*orfS*-*ombC-omaC-omcC* with *orfS* further decreased ferrihydrite reduction. At 360 h, *G. sulfurreducens* PCA and Δ*ombB-omaB-omcB*-*orfS*-*ombC-omaC-omcC* with the empty vector reduced 22.1 ± 1.4 and 5.5 ± 0.5 mM ferrihydrite (*n* = 3), respectively. Complement of Δ*ombB-omaB-omcB*-*orfS*-*ombC-omaC-omcC* with *orfS in trans* reduced only 2.7 ± 0.4 mM ferrihydrite (*n* = 3), which was very close to 2.0 ± 0.3 mM ferrihydrite (*n* = 3) reduced by Δ*B*/Δ*C* clusters with the empty vector (**Figure 4C**). Thus, the presence of *orfS* appears to negatively impact Fe(III) reduction by *G. sulfurreducens.*

## Discussion

In the presence of *ombC-omaC-omcC*, disruptions of *ombB*, *omaB*, *omcB*, or *ombB-omaB* had no impact on Fe(III)-citrate reduction by *G. sulfurreducens* PCA. These results are consistent with our previous finding that replacement of *ombB-omaB-omcB* with an antibiotic gene only slightly decreased Fe(III)-citrate reduction by *G. sulfurreducens* PCA (Liu et al., 2014). However, no observed phenotype for Δ*omcB* in Fe(III)-citrate reduction by *G. sulfurreducens* PCA contrasts with previous results for *G. sulfurreducens* DL-1, in which replacement of *omcB* significantly impaired this microorganism’s ability to reduce Fe(III)-citrate (Leang et al., 2003). This difference between *G. sulfurreducens* PCA and *G. sulfurreducens* DL-1 regarding the role of *omcB* in Fe(III)-citrate reduction was hypothesized to be attributed to the different ability of these two bacterial strains to compensate for the loss of *omcB* (Liu et al., 2014). In sharp contrast to that in the presence of *ombC-omaC-omcC*, disruptions of *ombB*, *omaB*, *omcB*, or *ombB-omaB* in the absence of *ombC-omaC-omcC* greatly impaired the bacterial ability to reduce Fe(III)-citrate as well as ferrihydrite. Together, these results unequivocally demonstrate that *ombC, omaC*, and *omcC* of *G. sulfurreducens* PCA can quickly compensate for the loss of their respective counterparts in *ombB-omaB-omcB*. These results are consistent with our previous findings that both *ombB-omaB-omcB* and *ombC-omaC-omcC* contributed to the extracellular reduction of Fe(III)-citrate and ferrihydrite by *G. sulfurreducens* PCA (Liu et al., 2014). They are also consistent with the findings that both *ombB-omaB-omcB* and *ombC-omaC-omcC* of *G. sulfurreducens* PCA are expressed under the conditions tested and they are highly identical (71–100% identical at the amino acid sequence level; Leang et al., 2003; Liu et al., 2014; Shi et al., 2014).

The observed overlapping role of *ombB-omaB-omcB* and *ombC-omaC-omcC* in Fe(III)-citrate and ferrihydrite reduction by *G. sulfurreducens* PCA is, however, different from that of *mtrD-mtrE-mtrF*-*omcA*-*mtrC-mtrA-mtrB* of *S. oneidensis* MR-1. Although *mtrA*/*mtrD*, *mtrB*/*mtrD*, and *mtrC*/*mtrF* are paralogs, respectively, only *mtrC-mtrA-mtrB* have been implicated in extracellular reduction of Fe(III) by *S. oneidensis* MR-1and the role of *mtrD-mtrE-mtrF* remains unclear (Hartshorne et al., 2009; Coursolle and Gralnick, 2010).

The reduction kinetics of Δ*ombB/*Δ*C* cluster, Δ*omaB/*Δ*C* cluster, Δ*omcB/*Δ*C* cluster, Δ*ombB-omaB/*Δ*C* cluster and Δ*B*/Δ*C* clusters are nearly identical. These results support previous findings that OmbB, OmaB, and OmcB form a functional protein complex, which is a 20-heme trimer, for transferring electrons across the outer membrane (Liu et al., 2014). Thus, loss of any subunit of this protein complex eliminates its function. This is, however, different from the MtrABC protein complex of *S. oneidensis* MR-1 in which MtrAB can still transfer electrons across the outer membrane in the absence of MtrC (Hartshorne et al., 2009; White et al., 2013). This difference between OmbB/OmaB/OmcB of *G. sulfurreducens* PCA and MtrABC of *S. oneidensis* MR-1 may be attributed to the fact that the periplasmic *c*-Cyt OmaB contains eight hemes and the outer-membrane *c*-Cyt OmcB has 12 hemes, while each of the periplasmic *c*-Cyt MtrA and outer-membrane *c*-Cyt MtrC possesses 10 hemes (Shi et al., 2005, 2006; Hartshorne et al., 2007; Liu et al., 2014). The eight hemes of OmaB may not form a heme-based conduit that is long enough to span the entire width of the outer membrane and may require the two hemes of OmcB for transferring electrons across the outer membrane. Consistent with this suggestion, OmcB is only partially exposed to the bacterial surface (Qian et al., 2007). It is, thus, possible that a substantial portion of OmcB is inserted into the porin-like outer-membrane protein OmbB where it interfaces with OmaB to facilitate transfer of electrons across the outer membrane, which is similar in principle to the MtrABC protein complex of *S. oneidensis* MR-1 (Hartshorne et al., 2009; Richardson et al., 2012). Alternatively, in *S. oneidensis* MR-1, Mtr-associated proteins, such as OmcA and MtrF, can compensate for the loss of MtrC (Coursolle and Gralnick, 2012), while in the absence of *ombC-omaC-omcC*, no additional outer membrane *c*-Cyt is available.

Our previous results showed that Δ*ombB-omaB-omcB*-*orfS*-*ombC-omaC-omcC* could still reduce Fe(III)-citrate and ferrihydrite although at a significantly decreased rate compared to the wild type. Because *G. sulfurreducens* PCA possessed two more pcc gene clusters in addition to *ombB-omaB-omcB* and *ombC-omaC-omcC*, we suggested that the residual ability of reducing Fe(III)-citrate and ferrihydrite by Δ*ombB-omaB-omcB*-*orfS*-*ombC-omaC-omcC* could be attributed to the functions of remaining pcc gene clusters or other redox proteins with trans-outer membrane electron transfer capabilities (Liu et al., 2014; Shi et al., 2014). The observed negative role of *orfS* in reducing Fe(III)-citrate and ferrihydrite by the mutant without *ombB-omaB-omcB* and *ombC-omaC-omcC* indeed supports our previous suggestion. OrfS is predicted to be a transcriptional factor of the TetR family. Previous results indicated that OrfS was not involved in regulating expression of *ombB-omaB-omcB* or *ombC-omaC-omcC* clusters and its functional role was unclear (Leang and Lovley, 2005). The results reported from this study clearly show that presence of *orfS* decreases Fe(III)-citrate and ferrihydrite reduction. Given that most members of the TetR family are transcriptional repressors (Ramos et al., 2005), we hypothesize that OrfS may also negatively regulate the expression of other genes with functions similar to that of *ombB-omaB-omcB* and *ombC-omaC-omcC* when Fe(III) serves as the terminal electron acceptor.

In summary, the results from this study clearly demonstrate the direct involvements of *ombB*, *ombB*, and *omcB* in extracellular reduction of Fe(III)-citrate and ferrihydrite by *G. sulfurreducens* PCA. They also show a negative role of *orfS* in extracellular reduction of Fe(III) by *G. sulfurreducens* PCA. Moreover, the results from this as well as previous investigations collectively demonstrate the involvement of *ombB-omaB-omcB*, *ombC-omaC-omcC* and probably other proteins in electron conductance across the outer membrane during extracellular reduction of Fe(III) by *G. sulfurreducens* PCA. Existence of multiple and parallel trans-outer membrane extracellular electron transfer pathways critical to extracellular reduction of Fe(III) not only reflects the importance of extracellular reduction of Fe(III) in the physiology of *G. sulfurreducens*, but also is probably one of the main reasons that the results of Fe(III) reduction by this bacterium from different research groups are sometimes not comparable.

## Author Contributions

JF, JZ, and LS designed the study. YL conducted the research. YL, JF, JZ, and LS wrote the paper.

## Conflict of Interest Statement

The authors declare that the research was conducted in the absence of any commercial or financial relationships that could be construed as a potential conflict of interest.

## References

[B1] Bird L. J. Bonnefoy V. Newman D. K. (2011). Bioenergetic challenges of microbial iron metabolisms. *Trends Microbiol.* 19 330–340. 10.1016/j.tim.2011.05.001 21664821

[B2] Bond D. R. Strycharz-Glaven S. M. Tender L. M. Torres C. I. (2012). On electon transport through Geobacter biofilms. *ChemSumChem* 5 1099–1105. 10.1002/cssc.201100748 22615023

[B3] Butler J. E. Young N. D. Lovley D. R. (2009). Evolution from a respiratory ancestor to fill syntrophic and fermentative niches: comparative genomics of six Geobacteraceae species. *BMC Genomics* 10:103 10.1186/1471-2164-10-103 PMC2669807 19284579

[B4] Chang A. C. Cohen S. N. (1978). Construction and characterization of amplifiable multicopy DNA cloning vehicles derived from the P15A cryptic miniplasmid. *J. Bacteriol.* 134 1141–1156. 149110 10.1128/jb.134.3.1141-1156.1978 PMC222365

[B5] Coppi M. V. Leang C. Sandler S. J. Lovley D. R. (2001). Development of a genetic system for *Geobacter sulfurreducens*. *Appl. Environ. Microbiol.* 67 3180–3187. 10.1128/AEM.67.7.3180-3187.2001 11425739 PMC92998

[B6] Coursolle D. Gralnick J. A. (2010). Modularity of the Mtr respiratory pathway of *Shewanella oneidensis* strain MR-1. *Mol. Microbiol.* 77 995–1008. 10.1111/j.1365-2958.2010.07266.x 20598084

[B7] Coursolle D. Gralnick J. A. (2012). Reconstruction of extracellular respiration pathways for iron(III) reduction in *Shewanella oneidensis* strain MR-1. *Front. Microbiol.* 3:56 10.3389/fmicb.2012.00056 PMC3282943 22363330

[B8] Hartshorne R. S. Jepson B. N. Clarke T. A. Field S. J. Fredrickson J. Zachara J. (2007). Characterization of *Shewanella oneidensis* MtrC: a cell-surface decaheme cytochrome involved in respiratory electron transport to extracellular electron acceptors. *J. Biol. Inorg. Chem.* 12 1083–1094. 10.1007/s00775-007-0278-y 17701062

[B9] Hartshorne R. S. Reardon C. L. Ross D. Nuester J. Clarke T. A. Gates A. J. (2009). Characterization of an electron conduit between bacteria and the extracellular environment. *Proc. Natl. Acad. Sci. U.S.A.* 106 22169–22174. 10.1073/pnas.0900086106 20018742 PMC2799772

[B10] Kovach M. E. Elzer P. H. Hill D. S. Robertson G. T. Farris M. A. Roop R. M. (1995). Four new derivatives of the broad-host-range cloning vector pBBR1MCS, carrying different antibiotic-resistance cassettes. *Gene* 166 175–176. 10.1016/0378-1119(95)00584-1 8529885

[B11] Leang C. Coppi M. V. Lovley D. R. (2003). OmcB, a c-type polyheme cytochrome, involved in Fe(III) reduction in *Geobacter sulfurreducens*. *J. Bacteriol.* 185 2096–2103. 10.1128/JB.185.7.2096-2103.2003 12644478 PMC151516

[B12] Leang C. Lovley D. R. (2005). Regulation of two highly similar genes, omcB and omcC, in a 10 kb chromosomal duplication in *Geobacter sulfurreducens*. *Microbiology* 151 1761–1767. 10.1099/mic.0.27870-0 15941985

[B13] Liu Y. Wang Z. Liu J. Levar C. Edwards M. J. Babauta J. T. (2014). A trans-outer membrane porin-cytochrome protein complex for extracellular electron transfer by *Geobactersul furreducens* PCA. *Environ. Microbiol. Rep.* 6 776–785. 10.1111/1758-2229.12204 25139405 PMC4282303

[B14] Lloyd J. R. Leang C. Hodges Myerson A. L. Coppi M. V. Cuifo S. (2003). Biochemical and genetic characterization of PpcA, a periplasmic c-type cytochrome in *Geobacter sulfurreducens*. *Biochem. J.* 369 153–161. 10.1042/bj20020597 12356333 PMC1223068

[B15] Lovley D. R. Holmes D. E. Nevin K. P. (2004). Dissimilatory Fe(III) and Mn(IV) reduction. *Adv. Microb. Physiol.* 49 219–286. 10.1016/S0065-2911(04)49005-5 15518832

[B16] Lovley D. R. Ueki T. Zhang T. Malvankar N. S. Shrestha P. M. Flanagan K. A. (2011). Geobacter: the microbe electric’s physiology, ecology, and practical applications. *Adv. Microb. Physiol.* 59 1–100. 10.1016/B978-0-12-387661-4.00004-5 22114840

[B17] Qian X. Reguera G. Mester T. Lovley D. R. (2007). Evidence that OmcB and OmpB of *Geobacter sulfurreducens* are outer membrane surface proteins. *FEMS Microbiol. Lett.* 277 21–27. 10.1111/j.1574-6968.2007.00915.x 17986080

[B18] Ramos J. L. Martinez-Bueno M. Molina-Henares A. J. Teran W. Watanabe K. Zhang X. (2005). The TetR family of transcriptional repressors. *Microbiol. Mol. Biol. Rev.* 69 326–356. 10.1128/MMBR.69.2.326-356.2005 15944459 PMC1197418

[B19] Richardson D. J. Butt J. N. Fredrickson J. K. Zachara J. M. Shi L. Edwards M. J. (2012). The ‘porin-cytochrome’ model for microbe-to-mineral electron transfer. *Mol. Microbiol.* 85 201–212. 10.1111/j.1365-2958.2012.08088.x 22646977

[B20] Rollefson J. B. Levar C. E. Bond D. R. (2009). Identification of genes involved in biofilm formation and respiration via mini-Himar transposon mutagenesis of *Geobacter sulfurreducens*. *J. Bacteriol.* 191 4207–4217. 10.1128/JB.00057-09 19395486 PMC2698481

[B21] Schwertman U. Cornell R. M. (2000). *Iron Oxides in the Laboratory: Preparation and Characterization.* Weinheim: Wiley-VCH.

[B22] Shi L. Chen B. Wang Z. Elias D. A. Mayer M. U. Gorby Y. A. (2006). Isolation of a high-affinity functional protein complex between OmcA and MtrC: Two outer membrane decaheme c-type cytochromes of *Shewanella oneidensis* MR-1. *J. Bacteriol.* 188 4705–4714. 10.1128/JB.01966-05 16788180 PMC1483021

[B23] Shi L. Fredrickson J. Zachara J. (2014). Genomic analyses of bacterial porin-cytochrome gene clusters. *Front. Microbiol.* 5:657 10.3389/fmicb.2014.00657 PMC4245776 25505896

[B24] Shi L. Lin J. T. Markillie L. M. Squier T. C. Hooker B. S. (2005). Overexpression of multi-heme C-type cytochromes. *BioTechniques* 38 297–299. 10.2144/05382PT01 15727136

[B25] Shi L. Richardson D. J. Wang Z. Kerisit S. N. Rosso K. M. Zachara J. M. (2009). The roles of outer membrane cytochromes of *Shewanella* and *Geobacter* in extracellular electron transfer. *Environ. Microbiol. Rep.* 1 220–227. 10.1111/j.1758-2229.2009.00035.x 23765850

[B26] Shi L. Rosso K. M. Clarke T. A. Richardson D. J. Zachara J. M. Fredrickson J. K. (2012a). Molecular underpinnings of Fe(III) oxide reduction by *Shewanella oneidensis* MR-1. *Front. Microbiol.* 3:50 10.3389/fmicb.2012.00050 PMC3279761 22363328

[B27] Shi Z. Zachara J. M. Shi L. Wang Z. Moore D. A. Kennedy D. W. (2012b). Redox reactions of reduced flavin mononucleotide (FMN), riboflavin (RBF), and anthraquinone-2,6-disulfonate (AQDS) with ferrihydrite and lepidocrocite. *Environ. Sci. Technol.* 46 11644–11652. 10.1021/es301544b 22985396

[B28] Shi L. Squier T. C. Zachara J. M. Fredrickson J. K. (2007). Respiration of metal (hydr)oxides by *Shewanella* and *Geobacter*: a key role for multihaem c-type cytochromes. *Mol. Microbiol.* 65 12–20. 10.1111/j.1365-2958.2007.05783.x 17581116 PMC1974784

[B29] Snider R. M. Strycharz-Glaven S. M. Tsoi S. D. Erickson J. S. Tender L. M. (2012). Long-range electron transport in *Geobacter sulfurreducens* biofilms is redox gradient-driven. *Proc. Natl. Acad. Sci. U.S.A.* 109 15467–15472. 10.1073/pnas.1209829109 22955881 PMC3458377

[B30] Stookey L. (1970). Ferrozine-a new spectrophotometric reagent for iron. *Anal. Chem.* 42 779–781. 10.1021/ac60289a016

[B31] Strycharz-Glaven S. M. Snider R. M. Guiseppi-Elie A. Tender L. M. (2011). On the electrical conductivity of microbial nanowires and biofilms. *Energy Environ. Sci.* 4 4366–4379. 10.1039/c1ee01753e

[B32] Thomas P. E. Ryan D. Levin W. (1976). An improved staining procedure for the detection of the peroxidase activity of cytochrome P-450 on sodium dodecyl sulfate polyacrylamide gels. *Anal. Biochem.* 75 168–176. 10.1016/0003-2697(76)90067-1 822747

[B33] Wang Z. Liu C. Wang X. Marshall M. J. Zachara J. M. Rosso K. M. (2008). Kinetics of reduction of Fe(III) complexes by outer membrane cytochromes MtrC and OmcA of *Shewanella oneidensis* MR-1. *Appl. Environ. Microbiol.* 74 6746–6755. 10.1128/AEM.01454-08 18791025 PMC2576718

[B34] Weber K. A. Achenbach L. A. Coates J. D. (2006). Microorganisms pumping iron: anaerobic microbial iron oxidation and reduction. *Nat. Rev. Microbiol.* 4 752–764. 10.1038/nrmicro1490 16980937

[B35] White G. F. Shi Z. Shi L. Wang Z. Dohnalkova A. C. Marshall M. J. (2013). Rapid electron exchange between surface-exposed bacterial cytochromes and Fe(III) minerals. *Proc. Natl. Acad. Sci. U.S.A.* 110 6346–6351. 10.1073/pnas.1220074110 23538304 PMC3631691

