# Supplementary material for: Direct involvement of ombB, omaB, and omcB genes in extracellular reduction of Fe(III) by Geobacter sulfurreducens PCA
Source: Front Microbiol. 2015 Oct 1;6:1075. doi: 10.3389/fmicb.2015.01075 (PMC4589669; doi:10.3389/fmicb.2015.01075)
Supplement: Supplementary file 1 [file Data_Sheet_1.DOCX]

**Supplemental figures**

**Figure legends**

**Figure S1. Analyses of gene replacement mutants by heme-staining after SDS-PAGE.** (**A**) Gene replacement mutants in the presence of *ombC-omaC-omcC*. PCA, *G.* *sulfurreducens* PCA; 1, Δ*omcB*; 2, Δ*omaB*; 3, Δ*ombB*; 4, Δ*ombB-omaB*; 5, Δ*B* cluster and S, SeeBlue Plus 2 Pre-stained standards (Invitrogen, Grand Island, NY, USA). PCA and Δ*B* cluster are included for comparison. (**B**) Gene replacement mutants in the absence of *ombC-omaC-omcC*. PCA, *G.* *sulfurreducens* PCA; 1, Δ*B/∆C* clusters; 2, Δ*ombB*-*omaB/∆C* cluster; 3, Δ*ombB/∆C* cluster; 4, Δ*omaB/∆C* cluster; 5, Δ*omcB/∆C* cluster; 6, *∆C* cluster; 7, *∆B* cluster, 8, Δ*ombB-omaB-omcB-orfS-∆ombC-omaC-omcC* and S, SeeBlue Plus 2 Pre-stained standards. PCA, *∆C* cluster*, ∆B* cluster and Δ*ombB-omaB-omcB-orfS-∆ombC-omaC-omcC* are included for comparison. The migration positions of OmaB/OmaC, OmcB and OmcC are indicated. About 10^4^ CFU were loaded in each lane.

**Figure S2**. **Complements in Fe(III)-citrate reduction**. The values plotted are the average 0.5 N HCl extractable Fe(II) measured at 48 hours for each strain from triplicate assays, respectively, and error bars are standard deviations.

**Figure S3.** **Analyses of complement strains by heme-staining after SDS-PAGE.** Complement strains of Δ*ombB-omaB-omcB-orfS-ombC-omaC-omcC* with *ombB, omaB, ombB-omaB and omcB*. *G.* *sulfurreducens* PCA cells were used as a positive control. OmaB/OmaC and OmcB/OmcC are indicated. About 10^4^ CFU were loaded in each lane.
