# Supplementary material for: Direct involvement of ombB, omaB, and omcB genes in extracellular reduction of Fe(III) by Geobacter sulfurreducens PCA
Source: Front Microbiol. 2015 Oct 1;6:1075. doi: 10.3389/fmicb.2015.01075 (PMC4589669; doi:10.3389/fmicb.2015.01075)
Supplement: Supplementary file 2 [file Table_1.DOC]

Table S1. Bacterial strains, plasmids and primers used in this study

| **Strain, plasmid or primer** | **Relevant genotype, phenotype, description, or sequence (5’ to 3’)** | **Source, reference, or purpose** |
| --- | --- | --- |
| **Strains** |  |  |
| *G. sulfurreducens* PCA | Wild type | ATCC |
| LS1500 | Δ*ombB-omaB-omcB*, Km^ra^ | Liu et al., 2014 |
| LS1501 | Δ*ombC-omaC-omcC*, Km^r^ | Liu et al., 2014 |
| LS1502 | Δ*ombB-omaB-omcB-orfS-ombC-omaC-omcC*, Km^r^ | Liu et al., 2014 |
| LS1508 | pBBR1-MCS5 in LS1502, Km^r^ Gm^rb^ | Liu et al., 2014 |
| LS1509 | Δ*ombB*, Km^r^ | This study |
| LS1510 | Δ*omaB*, Km^r^ | This study |
| LS1511 | Δ*omcB*, Cm^rc^ | This study |
| LS1512 | Δ*ombB-omaB,* Km^r^ | This study |
| LS1513 | Δ*ombB/∆ombC-omaC-omcC,* Km^r^ Cm^r^ | This study |
| LS1514 | Δ*omaB/∆ombC-omaC-omcC*, Km^r^ Cm^r^ | This study |
| LS1515 | Δ*omcB/∆ombC-omaC-omcC*, Km^r^ Cm^r^ | This study |
| LS1516 | Δ*ombB-omaB/∆ombC-omaC-omcC*, Km^r^ Cm^r^ | This study |
| LS1517 | Δ*ombB-omaB-omcB/∆ombC-omaC-omcC*, Km^r^ Cm^r^ | This study |
| LS1518 | pLS500 in LS1513, Km^r^ Cm^r^ Gm^r^ | This study |
| LS1519 | pLS501 in LS1514, Km^r^ Cm^r^ Gm^r^ | This study |
| LS1520 | pLS502 in LS1515, Km^r^ Cm^r^ Gm^r^ | This study |
| LS1521 | pLS503 in LS1516, Km^r^ Cm^r^ Gm^r^ | This study |
| LS1522 | pLS504 in LS1517, Km^r^ Cm^r^ Gm^r^ | This study |
| LS1523 | pBBR1-MCS5 in LS1513, Km^r^ Cm^r^ Gm^r^ | This study |
| LS1524 | pBBR1-MCS5 in LS1514, Km^r^ Cm^r^ Gm^r^ | This study |
| LS1525 | pBBR1-MCS5 in LS1515, Km^r^ Cm^r^ Gm^r^ | This study |
| LS1526 | pBBR1-MCS5 in LS1516, Km^r^ Cm^r^ Gm^r^ | This study |
| LS1527 | pBBR1-MCS5 in LS1517, Km^r^ Cm^r^ Gm^r^ | This study |
| LS1528 | pLS505 in LS1502, Km^r^ Gm^r^ | This study |
|  |  |  |
| ***E. coli*** |  |  |
| Top10 | Host for cloning | Invitrogen |
| WM3064 | Donor strain for conjugation | W. Metcalf, Univ. of Illinois, Urbana |
|  |  |  |
| **Plasmids** |  |  |
| pBBR1-MCS2 | Kanamycin resistant gene, Km^r^ | Kovach et al., 1995 |
| pBBR1-MCS5 | Empty vector, Gm^r^ | Kovach et al., 1995 |
| pACYC184 | Chloramphenicol resistant gene, Cm^r^ | Chang & Cohen, 1978 |
| pLS500 | *ombB*, Gm^r^ | This study |
| pLS501 | *omaB*, Gm^r^ | This study |
| pLS502 | *omcB*, Gm^r^ | This study |
| pLS503 | *ombB-omaB*, Gm^r^ | This study |
| pLS504 | *ombB-omaB-omcB*, Gm^r^ | Liu et al., 2014 |
| pLS505 | *orfS*, Gm^r^ | This study |
|  |  |  |
| **Primers** |  |  |
| OUF | CCGCAACCTGCAGTCGTGCC | Construction of LS1511 & LS1515 |
| OUR | GCTGTGTTGTGGGGTGAGCTC | Construction of LS1511 & LS1515 |
| ODF | GGGTGCAGCGTTCAACGCC | Construction of LS1511, LS1515 & LS1517 |
| ODR | GCAACTCATGAGCTAATGGG | Construction of LS1511, LS1515 & LS1517 |
| OCF | CAGAAGAGCTCACCCCACAACACGGAAGATCACTTCGC | Construction of LS1511 & LS1515 |
| OCR | GGCGTTGAACGCTGCACCCAGGGCACCAATAACTGCC | Construction of LS1511 , LS1515 & LS 1517 |
| BUF | TCGATCTACTCCGTGTTCGC | Construction of LS1510 & LS 1514 |
| BUR | GGATTTCCCCCCTTTCTAGA | Construction of LS1510 & LS 1514 |
| BDF | TTGAGAGCACCACACACCAC | Construction of LS1510, LS 1512, LS1514 & LS1516 |
| BDR | CGATATTGGAGAGAACGGCG | Construction of LS1510, LS 1512, LS1514 & LS1516 |
| BKF | TCTAGAAAGGGGGGAAATCCAGCGAACCGGAATTGCCAGCT | Construction of LS1510, |
| BKR | GTGGTGTGTGGTGCTCTCAATCAGAAGAACTCGTCAAGAAGGC | Construction of LS1510 & LS 1512 |
| AUF | TGGCCCGGAAATGTAAGCAA | Construction of LS1509, LS1512, LS1513, LS1516 & LS1517 |
| AUR | CATTAAGTGGACCTCCTTTC | Construction of LS1509, LS1512, LS1513, LS1516 & LS1517 |
| ADF | CGACTTCTAGAAAGGGGGGA | Construction of LS1509 & LS1513 |
| ADR | TTCTGATATTGAGCAATGAG | Construction of LS1509 & LS1513 |
| AKF | GAAAGGAGGTCCACTTAATGAGCGAACCGGAATTGCCAGCT | Construction of LS1509 & LS1512 |
| AKR | TCCCCCCTTTCTAGAAGTCGTCAGAAGAACTCGTCAAGAAGGC | Construction of LS1509 |
| BCF | TCTAGAAAGGGGGGAAATCCCGGAAGATCACTTCGC | Construction of LS1514 |
| BCR | GTGGTGTGTGGTGCTCTCAAAGGGCACCAATAACTGCC | Construction of LS1514 & LS1516 |
| ACF | GAAAGGAGGTCCACTTAATGCGGAAGATCACTTCGC | Construction of LS1513, LS1516 & LS1517 |
| ACR | TCCCCCCTTTCTAGAAGTCGAGGGCACCAATAACTGCC | Construction of LS1513 |
| OF | GATAAGCTTGGTACGTACTAATTGAGAGC (HindIII is underlined) | Construction of pLS502 |
| OR | TAAGGATCCGGAAAGCATGATCTGCTTGC(BamHI is underlined) | Construction of pLS502 |
| BF | GATCTCGAGGTGAAGAAATGGTTTATCGC (XhoI is underlined) | Construction of pLS501 |
| BR | GCGGAATTCTTAGTACGTACCAGGAAGGT (EcoRI is underlined) | Construction of pLS501 & pLS503 |
| AF | GCACTCGAGATGGGAATCAAAGGGTTCAC (XhoI is underlined) | Construction of pLS500, pLS503 & pLS504^1^ |
| AR | GCAGAATTCCTAGAAGTCGTAGTCAAAGA (EcoRI is underlined) | Construction of pLS500 |
| ABOR | TAAGAATTCGGAAAGCATGATCTGCTTGC (EcoRI is underlined) | Construction of pLS504^1^ |
| SF | GCACTCGAGGGGTGCAGCGTTCAACGCCA (XhoI is underlined) | Construction of pLS505 |
| SR | GCAACTAGTTAAGTGGACCTCCTTTCTTC-3’ (SpeI is underlined) | Construction of pLS505 |

^a^, kanamycin resistance; ^b^, gentamicin resistance; ^c^, chloramphenicol resistance.

**References**

Chang, A.C. and Cohen S. N. (1978). Construction and characterization of amplifiable multicopy DNA cloning vehicles derived from the P15A cryptic miniplasmid. *J. Bacteriol.* 134, 1141-1156.

Kovach, M. E., Elzer P. H., Hill D. S., Robertson G. T., Farris M. A., Roop R. M., et al., (1995). Four new derivatives of the broad-host-range cloning vector pBBR1MCS, carrying different antibiotic-resistance cassettes. *Gene* 166, 175-176.

Liu, Y., Wang Z., Liu J., Levar C., Edwards M. J., Babauta J. T., et al. (2014). A trans-outer membrane porin-cytochrome protein complex for extracellular electron transfer by *Geobacter sulfurreducens* PCA. *Environ. Microbiol. Rep.* 6, 776-785.
