# Supplementary figures and images for: Direct involvement of ombB, omaB, and omcB genes in extracellular reduction of Fe(III) by Geobacter sulfurreducens PCA
Source: Front Microbiol. 2015 Oct 1;6:1075. doi: 10.3389/fmicb.2015.01075 (PMC4589669; doi:10.3389/fmicb.2015.01075)

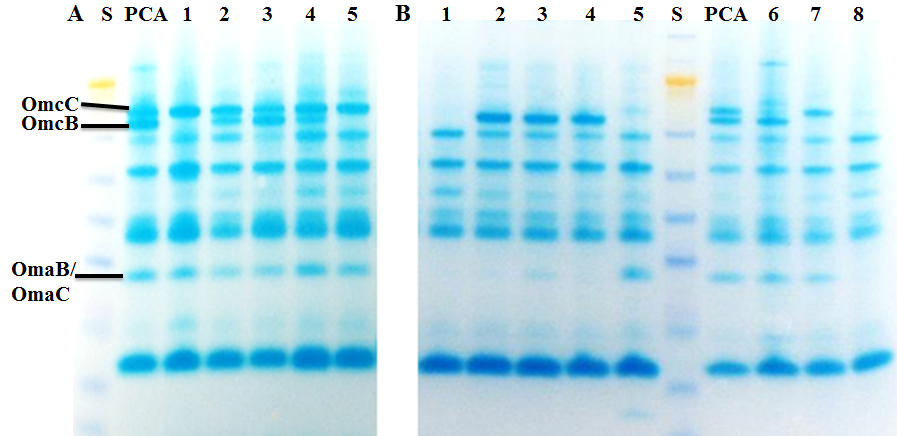

Supplement: Supplementary file 3 [file Image_1.TIF]

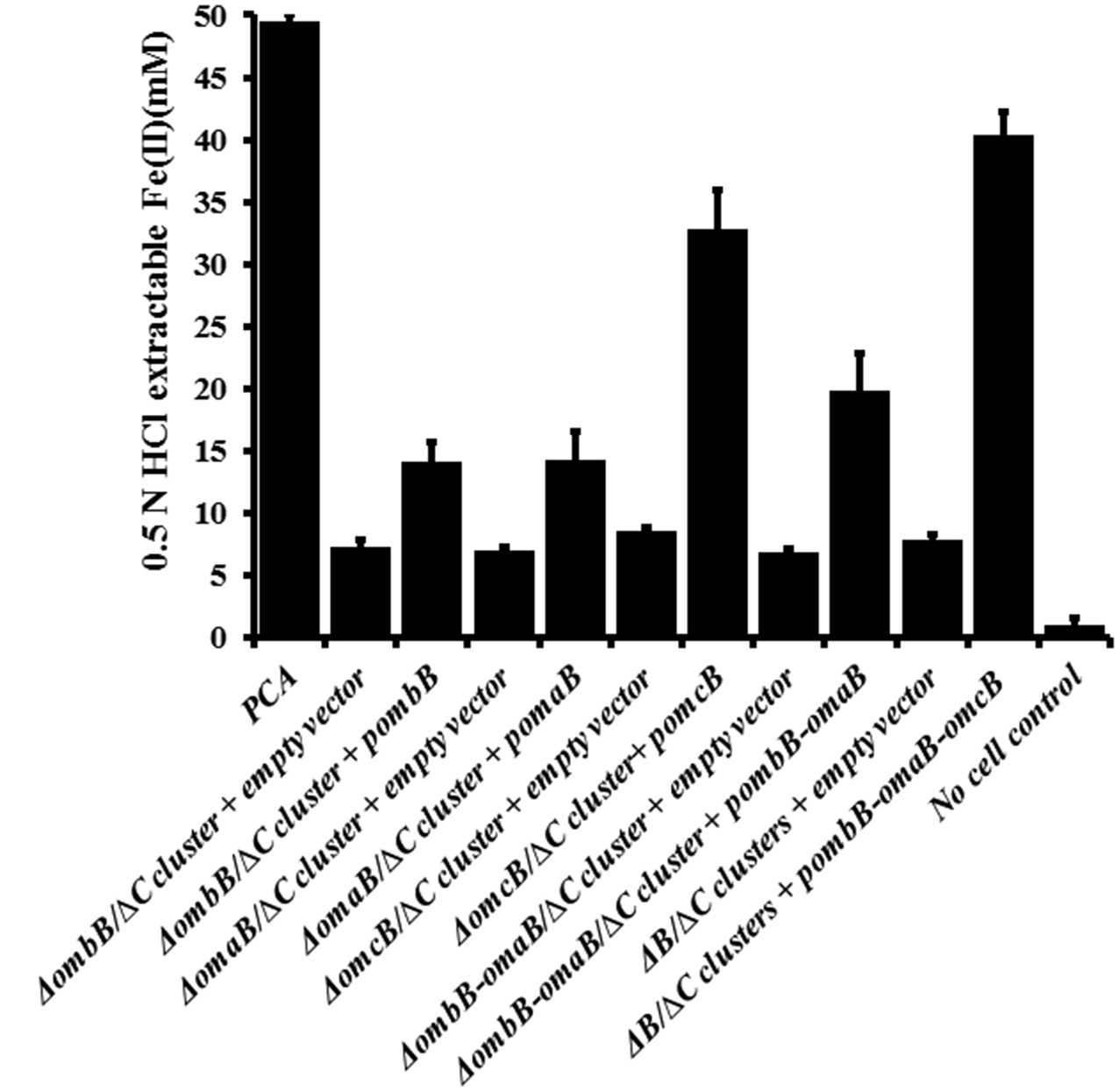

Supplement: Supplementary file 4 [file Image_2.TIF]

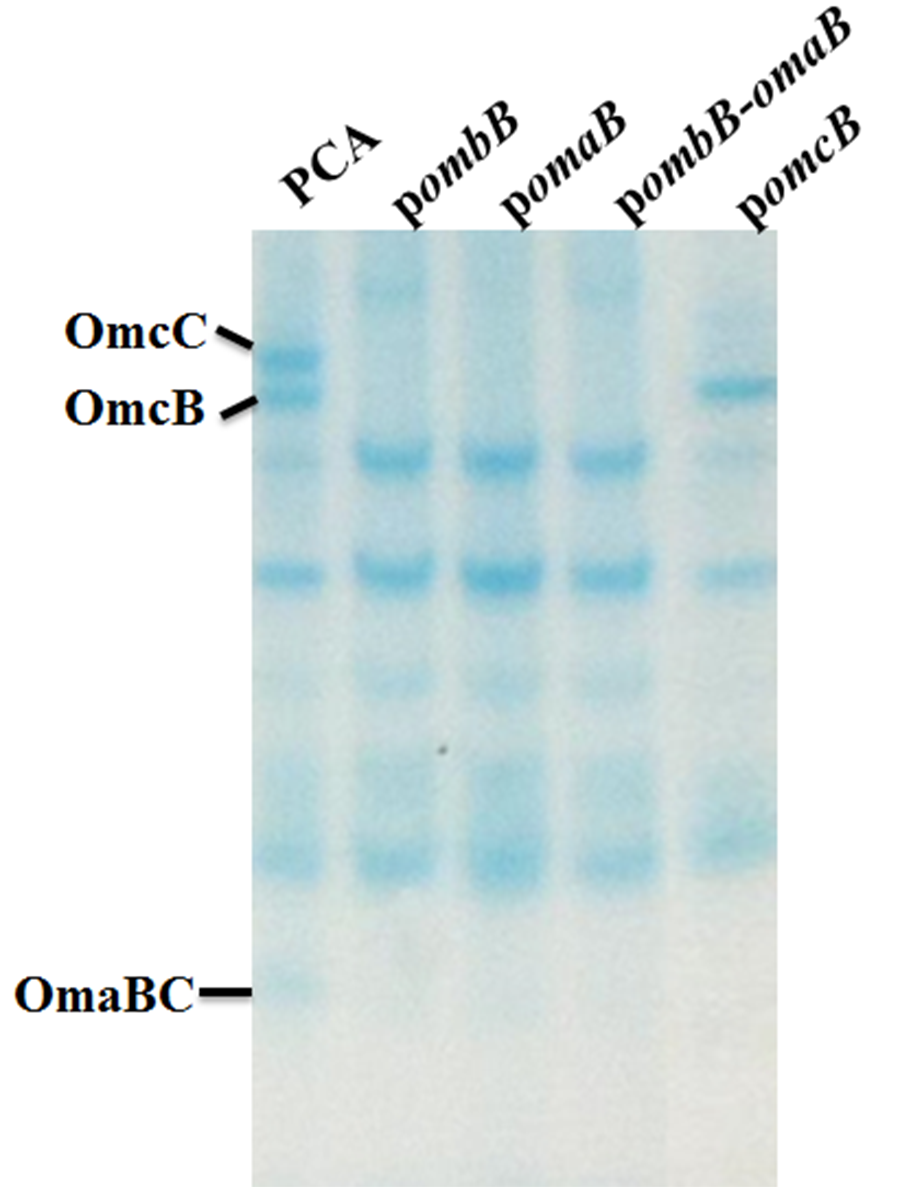

Supplement: Supplementary file 5 [file Image_3.TIF]
